# The Probationers' Forty-Eight-Hour Week

**Published:** 1920-01-10

**Authors:** 


### THE MATRONS' AND SISTERS' DEPARTMENT.

### THE PROBATIONERS' FORTY-EIGHT-HOUR WEEK.

Many are watching with some misgivings the progress of the agitation for shorter hours, and we hear in different quarters a plea for some elasticity in the system. Forty-eight hours may fit in well enough in some institutions. In others it is found that the fifty-six-hour week is well liked by the nurses. And in a great many fifty or fifty-two hours must be fixed unless a quite disproportionate increase of probationers takes place.

There can be no objection to variety except the fact that once it is admitted abuses begin to creep in. Where a standard has once been set managers, even the least expert, have a ready means of knowing whether or no they come up to it. The expressions "above the average," or "below the average," are liable to great abuse. Notoriously the guardians of ill-managed institutions take their stand on such averages. "You pay your nurses very badly," say the critics. "On the contrary I can assure you," says the Chairman glibly, "we pay them above the average for this class of institution." Or, "Your night nurses work eighty hours a week." "Yes," with a hypocritical sigh, "but the average for night nurses is eighty-four hours a week, and we are well below it." We greatly fear that until the hours which nurses ought to work are fixed by authority there will always be a long straggling group of institutions grading downwards on every kind of pretext from fair to unspeakably bad.

Meantime we regret the tendency to class together the hours probationers spend in getting the work done, and the hours they spend in improving their knowledge of nursing. It is idle to contend that all the work tells equally in the nurse's education. The usual routine of ward work consumes a long time every day. Unless a probationer be exceptionally stupid she learns to do parts of it as well as it can be done in a very short time. To go on doing things in which she is perfectly proficient because of the patients' needs, cannot be considered to advance her professional knowledge, though it has its distinct purpose in developing strength of character, patience, promptness, and other indispensable qualities. It is on this kind of work that a watch needs to be kept. It is not for the nurse's interests to be occupied too long over routine duties so that the learning processes, whether practical or theoretical, are unduly curtailed.

In badly organised training-schools great abuses used to occur, no doubt still do occur, in this direction. We used to hear of women in their second year cutting bread and butter or counting linen for months at a time. Many a nurse has bitter remembrances of having been left in some such backwater and watching the weeks go by without any sense of getting nearer to the goal of being a good nurse. From the point of view of training it is not so much hours of work as of uneducative work, which require to be shortened.

In educational centres a syllabus is drawn up for each pupil defining the number of available hours to be devoted to each subject. Some such plan is present to the mind of every capable matron when she blocks out her time-table, even if it be not stated in figures. The more efficient the training, the closer the grip on the way in which each probationer spends each of her forty-eight or fifty-six hours a week. Undoubtedly the heads of training-schools are in a very strong position, who are able to show their probationers that all extra time they put in over and above the forty-eight hours tells directly in the advancement either of their studies or their practical knowledge. To distinguish between the duty hours which are as it were a debt due to the hospital and the duty hours which are directly stimulating and educative for the probationer regarded as a nursing student is of the highest importance. The woman with a vocation learns much and very much in the former, and the woman without a vocation may perchance find it among the homely duties performed in the service of the sick. We have certainly no desire to belittle them. But the number of hours thus spent should not exceed a fair daily average prescribed by the matron who is guardian of the probationer's time.

To include all lectures and classes and study time for the probationer within the limits of the forty-eight hours assigned to her weekly work would not in our judgment advance her interests. If as must often happen demands have to be made on her to exceed the forty-eight hours week, let it be in this direction. Let her feel if possible that she is gaining an opportunity, whether in experience or in wider responsibility, by hours spent over and above those indicated in the schedule.

